# Editorial: Navigating intimacy & sexuality in the context of dementia

**DOI:** 10.3389/frdem.2024.1426327

**Published:** 2024-06-13

**Authors:** Cindy Jones, Maria Horne, Christine Brown Wilson

**Affiliations:** ^1^Faculty of Health Sciences and Medicine, Bond University, Gold Coast, QLD, Australia; ^2^Menzies Health Institute Queensland, Griffith University, Nathan, QLD, Australia; ^3^Faculty of Medicine and Health, School of Healthcare, University of Leeds, Leeds, United Kingdom; ^4^School of Nursing and Midwifery, Queens University, Belfast, United Kingdom

**Keywords:** dementia, aged care, sexuality, intimacy, sexual wellness

## 1 Introduction

Intimacy and sexuality in humans are important for psychological wellbeing and quality of life for humans. Such intimate and sexual needs are also pivotal in shaping our identities and relationships. These aspects of human experience do not fade with age or cognitive decline. Instead, they evolve, presenting unique challenges and opportunities for older individuals living with functional decline. The growing recognition of these challenges underscores the need for research to develop and inform caregiving practices and policy frameworks that acknowledge, respect and support the intimate and sexual rights and needs of older individuals living with dementia. The Research Topic “*Navigating intimacy & sexuality in the context of dementia*” in Frontiers in Dementia: Dementia Care highlights the multifaceted impact of dementia on intimate relationships and sexual expression. This Research Topic focused on providing insights and strategies that foster respectful and dignified care environments. This Research Topic aimed to chart a course toward compassionate and person-centered care by examining the expression of intimacy and sexuality, the prevailing stigmas around older adults' intimacy and sexuality needs, healthcare professionals and caregiver education to support such needs among older adults, the assessment of intimacy and sexuality needs, as well as the sexual rights of older individuals, living with dementia.

## 2 The impetus for change: unveiling the challenges

Dementia introduces significant changes in an older individual's sexual identity, relationship needs, orientation, and disposition, exacerbated by communication difficulties and increased vulnerability due to cognitive impairments. In addition, the stigma surrounding intimacy and sexuality in older adults, coupled with a lack of knowledge and training among healthcare professionals and caregivers, often leads to neglect or inappropriate responses to matters of intimacy and sexuality in dementia care. Such neglect not only undermines the dignity of older individuals living with dementia but also denies them their fundamental human rights. Recognition of the diverse ways in which older individuals living with dementia express intimacy, relationship needs, and sexuality is important. A review by Hafford-Letchfield on the emerging research into the lives of Lesbian, Gay, Bisexual, Transgender, Queer (or Questioning; LGBTQ+) older people living with dementia underscores the need and importance of sensitive care responses to their distinct needs; this area of research is still lacking in understanding and depth. The paper outlines a UK framework designed to improve care practices for the LGBTQ+ older population, emphasizing the education and training needs to encourage comprehensive and inclusive care approaches.

## 3 Forging ahead: developing responsive care strategies

The development and implementation of care strategies that support the intimate and sexual needs of older individuals with dementia requires a multi-dimensional approach. This includes the exploration of both psychosocial and pharmacological interventions, enhancing healthcare professionals and caregiver education and training, and advocating for the sexual rights of older individuals living with dementia. Moreover, creating supportive environments that prioritize privacy, autonomy, and respect for individual preferences is paramount. Horne et al. adopted a community-based participatory approach, to address these sensitive matters. Through participatory workshops involving care home staff, residents, and their close relations, they effectively developed educational and training materials focused on intimacy, sexuality and relationship needs. The study outcomes highlighted and encouraged the need for a co-production effort to produce resources that align and support the relationship, intimacy and sexuality needs and preferences of older care home residents by care staff, thereby supporting person-centered care.

## 4 Innovations in practice: the ISEP tool and the IPB scale

The introduction of tools like the Intimacy and Sexuality Expression Preference (ISEP) tool (Jones et al.) and the Including Personal Boundaries (IPB) scale (Waterschoot et al.) represents significant advancements in the field. The ISEP tool, for instance, facilitates meaningful conversations about intimacy and sexuality needs and preferences between healthcare professionals and older individuals, including those living with dementia. This user-centric approach offers a way to enhance care practices, ensuring the intimacy and sexuality needs and preferences of older individuals in aged care facilities are considered and addressed with dignity. Similarly, the IPB scale, developed through a co-creative process, offers a robust measure for assessing nurses' self-efficacy in managing intimate and sexual behaviors of older individuals living with dementia in nursing homes. This scale underlines the importance to respect personal boundaries and underscores the need for further research and education to provide caregivers with the skills necessary to navigate these complex situations effectively.

## 5 Conclusion: toward a future of dignified care

Understanding and supporting the intimate and sexual needs of older individuals living with dementia, while fraught with challenges, also contained opportunities for transformative change. As highlighted in this editorial, it is imperative to foster a care culture that is inclusive, respectful, and person-centered. Through continued research, education, and policy advocacy, we can pave the way for care environments where older individuals living with dementia can express their intimate, sexual and relationship needs and preferences without fear of stigma or neglect. In doing so, we not only uphold their dignity and rights but also enrich our collective understanding of human health and wellbeing regardless of age or cognitive decline, thereby improving quality of life.

## Author contributions

CJ: Writing – review & editing, Writing – original draft. MH: Writing – review & editing, Writing – original draft. CB: Writing – review & editing, Writing – original draft.

